# Complete genome sequence data of *Bacillus pumilus* GLB197, an effective antagonist of grape downy mildew

**DOI:** 10.1016/j.dib.2020.105423

**Published:** 2020-04-03

**Authors:** Qingchao Zeng, Jianbo Xie, Xun Zhang, Yan Li, Qi Wang

**Affiliations:** a College of Plant Protection, Key Laboratory of Plant Pathology, Ministry of Agriculture, China Agricultural University, Beijing, China; b College of Biological Sciences and Technology, Key Laboratory of Genetics and Breeding in Forest Trees and Ornamental Plants, Ministry of Education, Beijing Forestry University, Beijing, China; c Key Laboratory of Forest Protection, Research Institute of Forest Ecology, Environment and Protection, Chinese Academy of Forestry, State Forestry Administration, Beijing, China

**Keywords:** *Bacillus*, Genome Sequence, Biocontrol and Grape Downy Mildew

## Abstract

*Bacillus pumilus* GLB197 isolated from grape leaves exhibited strong inhibitory activity against grape downy mildew. The whole genome of the strain was sequenced to gain new insights into its molecular mechanism underlying the biocontrol on phytopathogens. The complete genome contains one chromosome (3,733,835 bp) and one plasmid (7061 bp). Several gene clusters related to biosynthesis of antimicrobial compounds were predicted. The genome provides insights into the possible biocontrol mechanisms and further application of this specific bacterium.

Specification table

**Table:** 

Subject area	Biology
More specific subject area	Microbiology and Genomics
Type of data	Complete genome sequence data of *Bacillus pumilus* GLB197
How data was acquired	Genome sequencing using PacBio RS Ⅱ and Illumina HiSeq at Tianjin Biochip Co., Ltd, China
Data format	Raw and analyzed data
Parameters for data collection	DNA was extracted from *B. pumilus* GLB197
Description of data collection	Whole genome sequencing, assembly, and annotation.
Data source location	*B. pumilus* GLB197 was isolated from the grape leaves,China.
Data accessibility	The genome sequence of *B. pumilus* GLB197 has been deposited in DDBJ/ENA/GenBank under the accession number CP018574 (https://www.ncbi.nlm.nih.gov/nuccore/CP018574) and CP018575 (https://www.ncbi.nlm.nih.gov/nuccore/CP018575).

## Value of the data

• The genome data of *B. pumilus* GLB197 may be helpful in understanding biological traits related to biocontrol against plant pathogens.
• The genome sequence of *B. pumilus* GLB197 provides fundamental knowledge of this organism and insight for biotechnological application in agriculture.
• The genome data of *B. pumilus* GLB197 will provide valuable information to perform comparative genomics analysis.

## 1 Data Description

To decrease the pesticide residue and environmental pollution in agricultural production, more and more bacteria are being applied as biological agents to suppress plant pathogens and promote plant growth [1], [2], [3]. Many antagonistic bacteria play fundamental roles in the sustainability natural ecosystem, and some of them can be used as inoculants to benefit plant growth and health [4,5]. In recent years, *B. pumilus* has been used as a biocontrol agent for protecting crops against fungal disease. For example, *B. pumilus* INR7 is an endophytic bacterium that has been commercialized as a biocontrol product against soil-borne and foliar pathogens [6]. *B. pumilus* GM3FR isolated from aerial plant tissues are used as biocontrol agents against phytopathogens [7].

Recently, we isolated strain GLB197 from grape leaves. This strain exhibits strong inhibitory effect on the growth of *Plasmopara viticola*, a fungal pathogen causing grape downy mildew. The strain is assigned to *B. pumilus* based on 16S rRNA and *gyrB* sequence analyses [8]. The potential mechanisms of action of *B. pumilus* GLB197, such as inhibition of the growth of plant pathogens, should be explored further. To gain knowledge on the genetic equipment of this bacterium and provide insight into the mechanism by which it plays its biocontrol roles, we sequenced and annotated the complete genome sequence of the strain. The complete genome sequence of *B. pumilus* GLB197 is composed of two replicons, a circular chromosome of 3,733,835 bp with a mean G + C content of 41.56%. Strain GLB197 harbors a plasmid of 7061 bp with G + C content of 35.14%, which is lower than that in the chromosomes. The chromosome contains 3,770 putative coding sequences, 80 tRNAs, 24 rRNAs, and 1 tmRNA. Six gene clusters were predicted to be responsible for the antimicrobial activity of *B. pumilus* GLB197. The GLB197 genome contains one gene clusters of nonribosomal peptide synthetase, such as lichenysin, for antibiotic production. The lichenysin cluster may be responsible for the inhibition of grape downy mildew and needs to be further studied. The complete genome data will be helpful to understand the molecular mechanisms of biocontrol of *B. pumilus* GLB197 and are beneficial for development of microbial fertilizers or biocontrol agents to improve crop production.

## 2 Experimental Design, Materials, and Methods

A high-quality genomic DNA was extracted, randomly fragmented, and then sequenced using the Illumina HiSeq and Pacific Biosciences (PacBio) platforms. A total of 1,397 M of sequence was generated from a 300-bp paired-end library, giving 349.25-fold coverage of the genome. Meanwhile, 604 M of sequences were obtained from a PacBio 10-kb library. Adaptor sequence removal, trimming, error correction, and assembly were performed using HGAP software [9]. Annotation was performed using rapid prokaryotic genome annotation software Prokka [10]. Putative proteins were searched against the COG, Gene Ontology, NCBI non-redundant protein database, and Kyoto Encyclopedia of Genes and Genomes (KEGG) databases. In addition, the secondary metabolite gene cluster was identified using the antiSMASHprogram [11].

## Appendix Supplementary materials

Image, application 1
